# Supplementary material for: Empirical methods for controlling false positives and estimating confidence in ChIP-Seq peaks
Source: BMC Bioinformatics. 2008 Dec 5;9:523. doi: 10.1186/1471-2105-9-523 (PMC2628906; doi:10.1186/1471-2105-9-523)
Supplement: Additional File 1 — A variety of html documents from the USeq web site detailing the available applications, their best usage, output file type descriptions, command line menus, etc. [file 1471-2105-9-523-S1.zip › USeqUserGuides/JavaDoc.zip!/JavaDoc/index-files/index-12.html]

 
 
 
 
 
 
M-Index
 


 

 
function windowTitle()
{
    parent.document.title="M-Index";
}
 
 
 

 

 


 
   
  
 
 
 
   
 
   
          Overview     
        Package   
        Class   
        Use   
          Tree     
          Deprecated     
       Index    
          Help     
   
 
 
  
 
 
 

 
  
   PREV LETTER   
   NEXT LETTER    
  
    FRAMES     
   NO FRAMES     
  
    All Classes  ');
  }
  //-->
 
 
    All Classes  
 


  
 
 
  
 

 A   B   C   D   E   F   G   H   I   J   L   M   N   O   P   Q   R   S   T   U   V   W   X   Z   
    
 M  
 
   main(String[])   - 
Static method in class bioroot. CronArchive 
  
   main(String[])   - 
Static method in class bioroot. DeleteMeNow 
  
   main(String[])   - 
Static method in class bioroot.parsers. ExtractWords 
  
   main(String[])   - 
Static method in class bioroot.parsers. RenameWords 
  
   main(String[])   - 
Static method in class bioroot. Spammer 
  
   main(String[])   - 
Static method in class cgar. DelmeTest 
  
   main(String[])   - 
Static method in class edu.utah.seq.analysis. DefinedRegionScanSeqs 
  
   main(String[])   - 
Static method in class edu.utah.seq.analysis. EnrichedRegionMaker 
  
   main(String[])   - 
Static method in class edu.utah.seq.analysis. QCSeqs 
  
   main(String[])   - 
Static method in class edu.utah.seq.analysis. ScanSeqs 
  
   main(String[])   - 
Static method in class edu.utah.seq.compression. CompressFasta 
  
   main(String[])   - 
Static method in class edu.utah.seq.data. DividePointData 
  
   main(String[])   - 
Static method in class edu.utah.seq.data. FilterPointData 
  
   main(String[])   - 
Static method in class edu.utah.seq.data. Simulator 
  
   main(String[])   - 
Static method in class edu.utah.seq.data. SubSamplePointData 
  
   main(String[])   - 
Static method in class edu.utah.seq.methylation454. ReadAlignParser 
  
   main(String[])   - 
Static method in class edu.utah.seq.methylation454. StatMethylationData 
  
   main(String[])   - 
Static method in class edu.utah.seq.misc. MakeUSeq 
  
   main(String[])   - 
Static method in class edu.utah.seq.parsers. ElandMultiParser 
  
   main(String[])   - 
Static method in class edu.utah.seq.parsers. ElandParser 
  
   main(String[])   - 
Static method in class edu.utah.seq.parsers. ElandSequenceParser 
  
   main(String[])   - 
Static method in class edu.utah.seq.parsers. JohnsonToTag 
  
   main(String[])   - 
Static method in class edu.utah.seq.parsers. Tag2Point 
  
   main(String[])   - 
Static method in class expr. CorrelationMaps 
  
   main(String[])   - 
Static method in class expr. ExpressionScores2CoordinatesViaGFF 
  
   main(String[])   - 
Static method in class expr. FileCrossFilter 
  
   main(String[])   - 
Static method in class expr. FileMatchJoiner 
  
   main(String[])   - 
Static method in class expr. NormalizeAgilentData 
  
   main(String[])   - 
Static method in class expr. ParseAgilentData 
  
   main(String[])   - 
Static method in class expr. ParseManipulateColumnDataForGeneSifter 
  
   main(String[])   - 
Static method in class expr. ParseMiRNATable 
  
   main(String[])   - 
Static method in class expr. ParseWormMart 
  
   main(String[])   - 
Static method in class expr. StripZeros 
  
   main(String[])   - 
Static method in class gata.aligner. DelmeAligner 
  
   main(String[])   - 
Static method in class gata.aligner. GATAligner 
  
   main(String[])   - 
Static method in class gata.main. GATAPlotter 
  
   main(String[])   - 
Static method in class igb.util. HistogramSgrOrGrFile 
  
   main(String[])   - 
Static method in class igb.util. Windows2HeatMapSgr 
  
   main(String[])   - 
Static method in class meme. MemeR 
  
   main(String[])   - 
Static method in class selex. SelexSeqParser 
  
   main(String[])   - 
Static method in class trans.anno. AnnotateRegions 
  
   main(String[])   - 
Static method in class trans.anno. AnnotateRegionsWithGeneList 
  
   main(String[])   - 
Static method in class trans.anno. BindingRegionsToGff3 
  
   main(String[])   - 
Static method in class trans.anno. BindingRegionsToIntervals 
  
   main(String[])   - 
Static method in class trans.anno. DmelCGNameCoordinatePrinter 
  
   main(String[])   - 
Static method in class trans.anno. FindNeighboringGenes 
  
   main(String[])   - 
Static method in class trans.anno. Gff2ToBindingRegions 
  
   main(String[])   - 
Static method in class trans.anno. Gff2ToIntervals 
  
   main(String[])   - 
Static method in class trans.anno. IntersectBindingPeaks 
  
   main(String[])   - 
Static method in class trans.anno. IntersectKeyWithRegions 
  
   main(String[])   - 
Static method in class trans.anno. IntersectRegions 
  
   main(String[])   - 
Static method in class trans.anno. MergeRegions 
  
   main(String[])   - 
Static method in class trans.anno. RandomRegions 
  
   main(String[])   - 
Static method in class trans.anno. SubtractRegions 
  
   main(String[])   - 
Static method in class trans.bkgrnd. CalcBkgrndRatios 
  
   main(String[])   - 
Static method in class trans.bkgrnd. CalculateBackgroundStats 
  
   main(String[])   - 
Static method in class trans.bkgrnd. MapControlIntensities 
  
   main(String[])   - 
Static method in class trans.cel. AverageACels 
  
   main(String[])   - 
Static method in class trans.cel. AveragePCels 
  
   main(String[])   - 
Static method in class trans.cel. CelFileConverter 
  
   main(String[])   - 
Static method in class trans.cel. CelPHistogram 
  
   main(String[])   - 
Static method in class trans.cel. CelProcessor 
  
   main(String[])   - 
Static method in class trans.cel. ConvertTVSToCelps 
  
   main(String[])   - 
Static method in class trans.cel. MakeChromosomeSets 
  
   main(String[])   - 
Static method in class trans.cel. QuantileNormalization 
  
   main(String[])   - 
Static method in class trans.cel. RotateCelFile 
  
   main(String[])   - 
Static method in class trans.cel. SplineScalar 
  
   main(String[])   - 
Static method in class trans.cel. StatIntensityFeatureArray 
  
   main(String[])   - 
Static method in class trans.graphics. AggregatePlots 
  
   main(String[])   - 
Static method in class trans.graphics. CelMasker 
  
   main(String[])   - 
Static method in class trans.graphics. CombineXYSeries 
  
   main(String[])   - 
Static method in class trans.graphics. IntervalPlotter 
  
   main(String[])   - 
Static method in class trans.graphics. PlotFloatArrays 
  
   main(String[])   - 
Static method in class trans.graphics. RankedSetAnalysis 
  
   main(String[])   - 
Static method in class trans.graphics. VirtualCel 
  
   main(String[])   - 
Static method in class trans.main. BindingRegionGraphPrinter 
  
   main(String[])   - 
Static method in class trans.main. ExportIntervalData 
  
   main(String[])   - 
Static method in class trans.main. FDRVsQValues 
  
   main(String[])   - 
Static method in class trans.main. FDRWindowConverter 
  
   main(String[])   - 
Static method in class trans.main. FindSubBindingRegions 
  
   main(String[])   - 
Static method in class trans.main. IntensityPrinter 
  
   main(String[])   - 
Static method in class trans.main. IntervalFilter 
  
   main(String[])   - 
Static method in class trans.main. IntervalGFFPrinter 
  
   main(String[])   - 
Static method in class trans.main. IntervalGraphPrinter 
  
   main(String[])   - 
Static method in class trans.main. IntervalMaker 
  
   main(String[])   - 
Static method in class trans.main. IntervalReportPrinter 
  
   main(String[])   - 
Static method in class trans.main. LoadChipSetIntervalOligoInfo 
  
   main(String[])   - 
Static method in class trans.main. LoadIntervalOligoInfo 
  
   main(String[])   - 
Static method in class trans.main. MakeAllWindows 
  
   main(String[])   - 
Static method in class trans.main. MergeWindowArrays 
  
   main(String[])   - 
Static method in class trans.main. MultiSGRPrinter 
  
   main(String[])   - 
Static method in class trans.main. MultiWindowIntervalMaker 
  
   main(String[])   - 
Static method in class trans.main. OligoIntensityPrinter 
  
   main(String[])   - 
Static method in class trans.main. OverlapCounter 
  
   main(String[])   - 
Static method in class trans.main. RandomizeLabels 
  
   main(String[])   - 
Static method in class trans.main. ReplaceWindowScores 
  
   main(String[])   - 
Static method in class trans.main. ScanChip 
  
   main(String[])   - 
Static method in class trans.main. ScanChromosomes 
  
   main(String[])   - 
Static method in class trans.main. ScanChromosomesCNV 
  
   main(String[])   - 
Static method in class trans.main. ScanGenes 
  
   main(String[])   - 
Static method in class trans.main. ScoreIntervals 
  
   main(String[])   - 
Static method in class trans.main. SetNumberIntervalMaker 
  
   main(String[])   - 
Static method in class trans.main. SplitWindowArraysByChromosome 
  
   main(String[])   - 
Static method in class trans.main. T2 
  
   main(String[])   - 
Static method in class trans.main. TestScanChip 
  
   main(String[])   - 
Static method in class trans.main. WilcoxonRankSumTest 
  
   main(String[])   - 
Static method in class trans.main. WilcoxonSignedRankTest 
  
   main(String[])   - 
Static method in class trans.main. WindowBlockMaker 
  
   main(String[])   - 
Static method in class trans.main. WindowBlockMakerTwoColor 
  
   main(String[])   - 
Static method in class trans.misc. AverageDoubles 
  
   main(String[])   - 
Static method in class trans.misc. Bar2Gr 
  
   main(String[])   - 
Static method in class trans.misc. Bed2Gr 
  
   main(String[])   - 
Static method in class trans.misc. BestWindowScoreExtractor 
  
   main(String[])   - 
Static method in class trans.misc. CalcGCAndTransform 
  
   main(String[])   - 
Static method in class trans.misc. CalcGCRatiosFromIntervals 
  
   main(String[])   - 
Static method in class trans.misc. CalculateGeneExtensions 
  
   main(String[])   - 
Static method in class trans.misc. ConvertAgilentData 
  
   main(String[])   - 
Static method in class trans.misc. ConvertBars2TPMapCel 
  
   main(String[])   - 
Static method in class trans.misc. ConvertGeoData 
  
   main(String[])   - 
Static method in class trans.misc. ConvertNimblegenNDF2TPMap 
  
   main(String[])   - 
Static method in class trans.misc. ConvertNimblegenPAIR2Cela 
  
   main(String[])   - 
Static method in class trans.misc. CorrelateGrFiles 
  
   main(String[])   - 
Static method in class trans.misc. Delme 
  
   main(String[])   - 
Static method in class trans.misc. Gr2Bar 
  
   main(String[])   - 
Static method in class trans.misc. InefficientPseudoMedian 
  
   main(String[])   - 
Static method in class trans.misc. PrependColumn 
 Prepends a column on columns of data.
   main(String[])   - 
Static method in class trans.misc. Regions2Intervals 
  
   main(String[])   - 
Static method in class trans.misc. ScoredRegionsFormatFixer 
  
   main(String[])   - 
Static method in class trans.misc. Sgr2Bar 
  
   main(String[])   - 
Static method in class trans.misc. TextPValOrSig2Gr 
  
   main(String[])   - 
Static method in class trans.misc. TransformRatios 
  
   main(String[])   - 
Static method in class trans.misc. Util 
  
   main(String[])   - 
Static method in class trans.misc. Wig2Bar 
  
   main(String[])   - 
Static method in class trans.misc. WindowHistogram 
  
   main(String[])   - 
Static method in class trans.qc. CelFileQualityControl 
  
   main(String[])   - 
Static method in class trans.qc. CoordinateExtractor1lq 
  
   main(String[])   - 
Static method in class trans.qc. DestypeIntensityCalculator 
  
   main(String[])   - 
Static method in class trans.qc. Parser1lq 
  
   main(String[])   - 
Static method in class trans.roc. ExonScoreExtractor 
  
   main(String[])   - 
Static method in class trans.roc. ExtractNonOverlappingWindows 
  
   main(String[])   - 
Static method in class trans.roc. ExtractSaveSgrScores 
  
   main(String[])   - 
Static method in class trans.roc. ExtractScoresForRoc 
  
   main(String[])   - 
Static method in class trans.roc. MethodTester 
  
   main(String[])   - 
Static method in class trans.roc. ParsePatternedWindows 
  
   main(String[])   - 
Static method in class trans.roc. ParseSgrsForParticularRegions 
  
   main(String[])   - 
Static method in class trans.roc. ParseSgrsForParticularScores 
  
   main(String[])   - 
Static method in class trans.roc. PWMScoreWindows 
  
   main(String[])   - 
Static method in class trans.roc. RocWindowScanner 
  
   main(String[])   - 
Static method in class trans.roc. ScoreParsedBars 
  
   main(String[])   - 
Static method in class trans.roc. SgrBacCounter 
  
   main(String[])   - 
Static method in class trans.tpmap. CollapseTPMap 
  
   main(String[])   - 
Static method in class trans.tpmap. ConvertTPMapsToFeatures 
  
   main(String[])   - 
Static method in class trans.tpmap. FilterTPMapByRegions 
  
   main(String[])   - 
Static method in class trans.tpmap. MummerMapper 
  
   main(String[])   - 
Static method in class trans.tpmap. OligoFlipper 
  
   main(String[])   - 
Static method in class trans.tpmap. Stat1lq 
  
   main(String[])   - 
Static method in class trans.tpmap. StatTPMap 
  
   main(String[])   - 
Static method in class trans.tpmap. StatTPMapControlSeqs 
  
   main(String[])   - 
Static method in class trans.tpmap. TPMapDensityReducer 
  
   main(String[])   - 
Static method in class trans.tpmap. TPMapDuplicateFilter 
  
   main(String[])   - 
Static method in class trans.tpmap. TPMapOligoBlastFilter 
  
   main(String[])   - 
Static method in class trans.tpmap. TPMapProcessor 
  
   main(String[])   - 
Static method in class trans.tpmap. TPMapSort 
  
   main(String[])   - 
Static method in class trans.tpmap. TPMapStripHighLowGC 
  
   main(String[])   - 
Static method in class trans.tpmap. WindowMaker 
  
   main(String[])   - 
Static method in class util.amazon. S3CodeSamples 
  
   main(String[])   - 
Static method in class util.amazon. S3Manager 
  
   main(String[])   - 
Static method in class util.apps. ArrayPrinter 
  
   main(String[])   - 
Static method in class util.apps. Correlate 
  
   main(String[])   - 
Static method in class util.apps. Delme2 
  
   main(String[])   - 
Static method in class util.apps. FileJoiner 
  
   main(String[])   - 
Static method in class util.apps. FileSplitter 
  
   main(String[])   - 
Static method in class util.apps. FileTrimmer 
  
   main(String[])   - 
Static method in class util.apps. FilterAgilentProbeFile 
  
   main(String[])   - 
Static method in class util.apps. Flash 
  
   main(String[])   - 
Static method in class util.apps. GeneCentricCNVParser 
  
   main(String[])   - 
Static method in class util.apps. GeneNameCounter 
  
   main(String[])   - 
Static method in class util.apps.gump. Gump 
  
   main(String[])   - 
Static method in class util.apps. HouseBean 
  
   main(String[])   - 
Static method in class util.apps. IntersectLists 
  
   main(String[])   - 
Static method in class util.apps. JQSub 
  
   main(String[])   - 
Static method in class util.apps. MakeBioTools 
  
   main(String[])   - 
Static method in class util.apps. MergeColumns 
 Merges the columns of two files using a tab, assumes the same length!
   main(String[])   - 
Static method in class util.apps. ParseMLSHTML 
  
   main(String[])   - 
Static method in class util.apps. ParserForGff 
  
   main(String[])   - 
Static method in class util.apps. PrintSelectColumns 
  
   main(String[])   - 
Static method in class util.apps. RenameRWSResults 
  
   main(String[])   - 
Static method in class util.apps. ScatterPlot 
  
   main(String[])   - 
Static method in class util.apps. ScoreChromosomes 
  
   main(String[])   - 
Static method in class util.apps. ScoreSequences 
  
   main(String[])   - 
Static method in class util.apps. SmoothData 
  
   main(String[])   - 
Static method in class util.apps. StatBarFiles 
  
   main(String[])   - 
Static method in class util.apps. SubtractOneFromEachColumn 
  
   main(String[])   - 
Static method in class util.apps. TASSignalChromFileSplitter 
  
   main(String[])   - 
Static method in class util.apps. TextParser 
  
   main(String[])   - 
Static method in class util.apps. WindowScoreSmoother 
  
   main(String[])   - 
Static method in class util.apps. ZipAndDelete 
  
   main(String[])   - 
Static method in class util.bio.annotation. ExportExons 
  
   main(String[])   - 
Static method in class util.bio.annotation. ExportIntergenicRegions 
  
   main(String[])   - 
Static method in class util.bio.annotation. ExportIntronicRegions 
  
   main(String[])   - 
Static method in class util.bio.cluster. HierarchicalClustering 
  
   main(String[])   - 
Static method in class util.bio.converters. AlignedBaseCounter 
  
   main(String[])   - 
Static method in class util.bio.converters. GenomeFASTASplitter 
  
   main(String[])   - 
Static method in class util.bio.converters. PossumToGFF 
  
   main(String[])   - 
Static method in class util.bio.converters. RepeatMaskerToGFF 
  
   main(String[])   - 
Static method in class util.bio.digest. REDigest 
  
   main(String[])   - 
Static method in class util.bio.parsers. ADFDataBaseSplitter 
  
   main(String[])   - 
Static method in class util.bio.parsers. BlastCompompositeGenerator 
  
   main(String[])   - 
Static method in class util.bio.parsers. BlastParser 
  
   main(String[])   - 
Static method in class util.bio.parsers. Cap3Parser 
  
   main(String[])   - 
Static method in class util.bio.parsers. DrosGeneCollFileParser 
  
   main(String[])   - 
Static method in class util.bio.parsers. FetchGenomicSequences 
  
   main(String[])   - 
Static method in class util.bio.parsers. FetchSeqsQualsByName 
  
   main(String[])   - 
Static method in class util.bio.parsers.gff. DmelRel4Extractor 
  
   main(String[])   - 
Static method in class util.bio.parsers.gff. ExtractGffFeatures 
  
   main(String[])   - 
Static method in class util.bio.parsers.gff. Gff3Parser 
  
   main(String[])   - 
Static method in class util.bio.parsers.gff. ParseWormBaseGffToUCSC 
  
   main(String[])   - 
Static method in class util.bio.parsers. ParseDecodeGenotypeData 
  
   main(String[])   - 
Static method in class util.bio.parsers. RefSeqAnnotationParser 
  
   main(String[])   - 
Static method in class util.bio.parsers. RipInparanoidResults 
  
   main(String[])   - 
Static method in class util.bio.parsers. RipMGIOrthologyResults 
  
   main(String[])   - 
Static method in class util.bio.parsers. ShiftPositionsInUCSCGeneLines 
  
   main(String[])   - 
Static method in class util.bio.parsers. SynonymMatching 
  
   main(String[])   - 
Static method in class util.bio.parsers. UCSCGeneModelTableReader 
  
   main(String[])   - 
Static method in class util.bio.seq. ConvertFasta2GCBoolean 
  
   main(String[])   - 
Static method in class util.bio.seq. ConvertFasta2RealSeqBoolean 
  
   main(String[])   - 
Static method in class util.bio.seq. FetchIndexedSequence 
  
   main(String[])   - 
Static method in class util.bio.seq. IndexFastas 
  
   main(String[])   - 
Static method in class util.bio.seq. LowerCase2Xs 
  
   main(String[])   - 
Static method in class util.bio.seq. MakeSpliceJunctionFasta 
  
   main(String[])   - 
Static method in class util.bio.seq. MakeTranscriptome 
  
   main(String[])   - 
Static method in class util.bio.seq. MaskExonsInFastaFiles 
  
   main(String[])   - 
Static method in class util.bio.seq. MergeFastas 
  
   main(String[])   - 
Static method in class util.bio.seq. OligoTiler 
  
   main(String[])   - 
Static method in class util.bio.seq. ParseSeqsFromUCSCGeneTable 
  
   main(String[])   - 
Static method in class util.bio.wrappers. Primer3Wrapper 
  
   main(String[])   - 
Static method in class util.bio.xamer. XamerCounter 
  
   main(String[])   - 
Static method in class util.gen. CubicSpline 
  
   main(String[])   - 
Static method in class util.gen. LinearRegression 
 An example.
   main(String[])   - 
Static method in class util.gen. MD5Crypt 
  
   main(String[])   - 
Static method in class util.gen. ReadZip 
  
   main(String[])   - 
Static method in class util.gen. SpearmanCorrelation 
  
   main(String[])   - 
Static method in class util.xml. ParseXMLFile 
 Starts XML parsing example
   makeAddOnePSPM(String[])   - 
Static method in class meme. MemeMotif 
 This method returns a position specific probability weight matrix given an array
 of Strings, each of the same size, containing GATC, no gaps, no N's.
   makeAlignmentObjects(ArrayList)   - 
Method in class gata.aligner. LocalAlignment 
  
   makeAllShapes(int, int)   - 
Method in class gata.plotter. AlignPanel 
  
   MakeAllWindows   - Class in  trans.main  Makes all windows given oligo positions.   MakeAllWindows(String[])   - 
Constructor for class trans.main. MakeAllWindows 
  
   makeAndLoadGeneExons()   - 
Method in class util.bio.parsers.gff. ParseWormBaseGffToUCSC 
  
   makeAndMoveIntervals()   - 
Method in class trans.main. T2 
 File management for intervals.
   makeAndSetAntibodyBeans(HttpSession, DBUtil, UserBean)   - 
Static method in class bioroot.antibody. AntibodySpreadSheet 
 Makes an array of AntibodyBeans based on Users AntibodyPreferences and sets them in the session.
   makeAndSetOligoBeans(HttpSession, DBUtil, UserBean)   - 
Static method in class bioroot.oligo. OligoSpreadSheet 
 Makes an array of OligoBeans based on Users OligoPreferences and sets them in the session.
   makeAndSetPlasmidBeans(HttpSession, DBUtil, UserBean)   - 
Static method in class bioroot.plasmid. PlasmidSpreadSheet 
 Makes an array of PlasmidBeans based on Users PlasmidPreferences and sets them in the session.
   makeAndSetStrainBeans(HttpSession, DBUtil, UserBean)   - 
Static method in class bioroot.strain. StrainSpreadSheet 
 Makes an array of StrainBeans based on Users StrainPreferences and sets them in the session.
   makeAnnotation(AnnoSpecParams)   - 
Method in class gata.main. GATAPlotter 
  
   makeAntibodyBeans(DBUtil, UserBean)   - 
Static method in class bioroot. Archive 
 Makes an array of all visibile AntibodyBeans
   makeArchive(DBUtil, UserBean)   - 
Static method in class bioroot. CronArchive 
 Returns null or a zip file containing a labGroup's full archive.
   makeArrayNames(float[][], float[][])   - 
Method in class trans.main. RandomizeLabels 
 Convert intensity arrays into an ArrayName[].
   makeArrowAndButt(int, int, int, double, int, double, double, double)   - 
Static method in class gata.main. GATAUtil 
 Makes an arrow and butt but no base line.
   makeArrowedTableRow(String[], String[])   - 
Static method in class util.gen. HTML 
 Example table row with arrow buttons.
   makeArrowLine(int, int, int, double, int, double, double, double)   - 
Static method in class gata.main. GATAUtil 
 Makes an arrow line with half arrow up for -1 or down for 1.
   makeArrowLine(int, int, int, float, int, float, float, float, float)   - 
Static method in class gata.main. GATAUtil 
 Makes an arrow line with half arrow up for -1 or down for 1.
   makeBarFileHashMap()   - 
Method in class trans.roc. ScoreParsedBars 
  
   makeBarFiles(Window[], File, int, boolean)   - 
Method in class trans.main. ScanChromosomes 
 Makes xxx.bar.zip files from a Window[], one per chromosome.
   makeBarFiles(Window[], File, int, boolean)   - 
Method in class trans.main. ScanChromosomesCNV 
 Makes xxx.bar.zip files from a Window[], one per chromosome.
   makeBaseCounts()   - 
Method in class util.bio.parsers. BlastCompompositeGenerator 
  
   makeBindingRegions(int[][], String)   - 
Static method in class trans.anno. IntersectRegions 
 Given an array of start stops and a chromosome, makes BindingRegions.
   makeBins()   - 
Method in class trans.roc. MethodTester 
  
   makeBins()   - 
Method in class trans.roc. SgrBacCounter 
  
   makeBins()   - 
Method in class util.gen. Histogram 
  
   MakeBioTools   - Class in  util.apps  Utility app to pull classes into folders and extract menus to html.   MakeBioTools()   - 
Constructor for class util.apps. MakeBioTools 
  
   makeBlankCela()   - 
Method in class trans.misc. ConvertBars2TPMapCel 
  
   makeBlastSummaryTable(BlastResult[])   - 
Static method in class bioroot. Search 
 Returns a summary HTML table, hotlinked id, name, score, expect
   makeBlockMapSgrFile(File)   - 
Method in class igb.util. Windows2HeatMapSgr 
 Takes an array of Window
   makeBufferedImage(double, boolean)   - 
Method in class gata.geneGlyphs. GlyphPanel 
  
   makeBufferedImage(double, boolean)   - 
Method in class gata.plotter. AlignPanel 
 Generates a BufferedImage from the panel.
   makeBufferedImage()   - 
Method in class trans.graphics. VirtualCelPanel 
  
   makeButton(String, int, ActionListener, JPanel)   - 
Static method in class gata.main. GATAUtil 
  
   makeButton(String)   - 
Method in class gata.plotter. ToolsFrame 
  
   makeButton(String)   - 
Method in class util.apps.gump. GumpToolPanel 
  
   makeButton(String, int, ActionListener, JPanel)   - 
Static method in class util.gen. Swing 
  
   makeByte4BaseMap()   - 
Static method in class util.bio.seq. Seq 
 Makes a hash map of each 4bp combination in the all4BaseCombinations String[] and a unique Byte.
   makeChallengeCard()   - 
Method in class util.gen. Passwords 
  
   makeCharts()   - 
Method in class trans.qc. CelFileQualityControl 
 Writes charts to the save directory and if so indicated displays them.
   makeChromosomeNameFileHash(File[])   - 
Static method in class util.bio.seq. Seq 
 Uses Seq.extractChromosomeName() to extract a chromosome name from each file.
   MakeChromosomeSets   - Class in  trans.cel      MakeChromosomeSets(String[])   - 
Constructor for class trans.cel. MakeChromosomeSets 
  
   makeChromosomeSets()   - 
Method in class trans.main. T2 
  
   makeChromosomeWideScores()   - 
Method in class trans.main. ScanChip 
 Makes chromosome wide scores.
   makeChromosomeWideScores()   - 
Method in class trans.main. ScanChromosomes 
 Makes chromosome wide scores.
   makeChromosomeWideScores()   - 
Method in class trans.main. ScanChromosomesCNV 
 Makes chromosome wide scores.
   makeChromSet(File[][], boolean)   - 
Method in class trans.main. T2 
  
   makeColors()   - 
Static method in class trans.graphics. VirtualCelPanel 
 Generates 768 diff colors along a black red yellow white gradient.
   makeConcatination()   - 
Method in class bioroot. Preference 
 Makes a visibility: orderBy: labGroups: features concat.
   makeConcatination()   - 
Method in class bioroot.strain. StrainPreference 
 Makes a visibility | orderBy | labGroups | features | strain types concat.
   makeConnectorLines()   - 
Method in class trans.graphics. RankedSetDrawPanel 
  
   makeConnectorLines()   - 
Method in class util.bio.cluster. ClusterDrawPanel 
 Builds tree and associated corr coeff JLabels.
   makeControlStats()   - 
Method in class trans.cel. QuantileNormalization 
 Instantiates a ControlStats object for each Quantile[] object.
   makeDataLines()   - 
Method in class trans.graphics. Graph 
  
   makeDirectories()   - 
Method in class trans.main. MakeAllWindows 
 Makes directories and files to hold point gr files and tempRatios
   makeDirectories()   - 
Method in class trans.main. ScanChromosomes 
 Makes directories and files to hold point gr files and tempRatios
   makeDirectories()   - 
Method in class trans.main. ScanChromosomesCNV 
 Makes directories and files to hold point gr files and tempRatios
   makeDoubleInputDialog(String, String, double)   - 
Static method in class gata.main. GATAUtil 
  
   makeDoubleInputDialog(String, String, double)   - 
Static method in class util.gen. Swing 
  
   makeDoublePercentInputDialog(String, String, double)   - 
Static method in class gata.main. GATAUtil 
  
   makeDoublePercentInputDialog(String, String, double)   - 
Static method in class util.gen. Swing 
  
   makeEditHistory(String, String, String)   - 
Static method in class bioroot. Util 
 Makes an edit history entry.
   makeEnrichedRegions()   - 
Method in class edu.utah.seq.analysis. EnrichedRegionMaker 
 Makes ERs given a window array.
   makeEnzymeArray(String, int)   - 
Method in class util.bio.digest. NEBParser 
  
   makeExonMask(UCSCGeneLine[])   - 
Method in class util.bio.annotation. ExportIntronicRegions 
  
   makeExons(int)   - 
Method in class trans.roc. ControlGene 
 Subtracts the endOffSet from the end and only creates an exon if 1+ end-start > 0
   makeExons(ControlGene[], int)   - 
Static method in class trans.roc. ControlGene 
 Fires makeExons on each CG within the ControlGene[].
   makeFastaAndFilter()   - 
Method in class trans.tpmap. MummerMapper 
 Makes a fasta file from a 1lq file, reverses the sequence of each oligo, drops control probes
   makeFeatures(String)   - 
Static method in class bioroot. Feature 
 Makes a Feature[] from a String concatinate, the thing that is actually stored in the database.
   makeFeatures(String[])   - 
Static method in class bioroot. Util 
 Use to make a default Feature[].
   makeField(String, AlignerInputPanel.NumberFocusListener)   - 
Method in class gata.aligner. AlignerInputPanel 
  
   makeField(JPanel, String, JFrame, JFileChooser, FileFocusListener)   - 
Static method in class gata.main. GATAUtil 
  
   makeFieldSimple(JPanel, String)   - 
Static method in class gata.main. GATAUtil 
  
   makeFieldSimple(JPanel, String)   - 
Static method in class util.gen. Swing 
  
   makeFileNameHash()   - 
Method in class meme. MemeResults 
  
   makeFinalReport()   - 
Method in class selex. SelexParams 
  
   makeFrequencyMatrix(String[])   - 
Static method in class meme. MemeMotif 
 Generates a matrix of the number of As Cc Gg Ts (top) by 1,2,3,4...positions in the
 motif (side) observed
 in all the Strings of the String[].
   makeFrequencyMatrix(String[])   - 
Static method in class util.bio.seq. Seq 
 Generates a matrix of the number of As Cc Gg Ts (top) by 1,2,3,4...positions in the
 motif (side) observed
 in all the Strings of the String[].
   makeFullPathName(String, String)   - 
Static method in class meme. UtilMeme 
  
   makeFullPathName(String, String)   - 
Static method in class util.gen. IO 
 Attempts to make a file and return it's canonical path name (full path name).
   makeGenericGlyphs()   - 
Method in class gata.geneGlyphs. Annotation 
 This makes a sorted array of GenericGlyphs broken down by track
   makeGlyphs()   - 
Method in class gata.geneGlyphs. Annotation 
 Call once to make the glyphs used in rendering the gene annotation.
   makeGlyphs(Graph, double)   - 
Method in class trans.graphics. Box 
  
   makeGlyphs(double, double, double, double)   - 
Method in class trans.graphics. Graph 
 Returns bottom y coordinate of graph.
   makeGlyphs()   - 
Method in class trans.graphics. RankedSetDrawPanel 
 Makes and places a GenomicRegionGlyph in each region using the rank number as the label.
   makeGridLines()   - 
Method in class trans.graphics. Graph 
  
   makeHashSelectStatement(String, HashMap, UserBean)   - 
Static method in class bioroot. Search 
 Makes an SQL SELECT statement using a HashMap.
   makeHeatMapBarFiles(Window[], File)   - 
Method in class trans.main. WindowBlockMaker 
 Be sure to set the scoreIndex, defaults to 0.
   makeHeatMapBarFiles(Window[], File, int)   - 
Method in class trans.main. WindowBlockMakerTwoColor 
  
   makeHeatMapBarFiles(Window[])   - 
Method in class trans.main. WindowBlockMakerTwoColor 
 Makes a stair step heat map from an array of windows in bar format.
   makeHeatMapGrFiles(Window[], File)   - 
Method in class trans.main. WindowBlockMaker 
 Be sure to set the scoreIndex, defaults to 0.
   makeHeatMapPositionValues(SmoothingWindow[], boolean)   - 
Method in class edu.utah.seq.data. HeatMapMaker 
 Returns a PointData object with loaded position and values representing a top score heatmap/ stairstep.
   makeHeatMapPositionValues(SmoothingWindow[])   - 
Method in class edu.utah.seq.data. HeatMapMakerPosNeg 
 Makes a stair step heat map from an array of windows in bar format.
   makeHeatMapSgrFile(Window[], File, boolean)   - 
Method in class trans.main. WindowBlockMaker 
 Be sure to set the scoreIndex, defaults to 0.
   makeHouseBeans()   - 
Method in class util.apps. ParseMLSHTML 
 Uses a hash to toss duplicate records, same mls#
   makeHugeTable()   - 
Static method in class util.gen. Passwords 
 An example in how to make a huge random word table.
   makeIndexedSequences()   - 
Method in class util.bio.seq. FetchIndexedSequence 
  
   makeInitialBGOs(File)   - 
Static method in class trans.bkgrnd. MapControlIntensities 
  
   makeInputDialog(String, String, String)   - 
Static method in class meme. MemeR 
  
   makeIntervals(File)   - 
Method in class trans.main. IntervalMaker 
  
   makeIntInputDialog(String, String, int)   - 
Static method in class gata.main. GATAUtil 
  
   makeIntInputDialog(String, String, int)   - 
Static method in class util.gen. Swing 
  
   makeLabel(JPanel, String, int, int)   - 
Static method in class gata.main. GATAUtil 
  
   makeLabel(String, int, int, double, double, double)   - 
Static method in class gata.main. GATAUtil 
  
   makeLabel(String)   - 
Method in class gata.plotter. ToolsFrame 
  
   makeLabel(String)   - 
Method in class util.bio.cluster. ClusterDrawPanel 
  
   makeLabel(JPanel, String, int, int)   - 
Static method in class util.gen. Swing 
  
   makeLabGroupPermissions()   - 
Method in class cgar.beans. UserBean 
 Finds available lab groups associated with this user.
   makeLibrary()   - 
Method in class edu.utah.seq.misc. MakeUSeq 
 Makes the code library jar from the directoriesForLibJar array.
   makeLibrary()   - 
Method in class util.apps. MakeBioTools 
 Makes the code library jar from the directoriesForLibJar array.
   makeLine(int, int, double, double, double)   - 
Static method in class gata.main. GATAUtil 
 Converts an int[2] (startStop) in nucleotides to a Line2D.Double
   makeLLPSPM(double[][], double, double, double, double)   - 
Static method in class meme. MemeMotif 
 takes a double[][] where array[0] refers to the first position in a motif
 array[0][0 to 3] = probability of A, C, G, or T nucleotide at that position.
   makeLocalAlignments(ArrayList)   - 
Method in class gata.aligner. Seqs 
  
   makeMaps()   - 
Method in class util.bio.seq. BinaryBooleanReaderWriter 
  
   makeMenuHTMLDoc()   - 
Method in class edu.utah.seq.misc. MakeUSeq 
 Generates the cmdLnMenus.html doc.
   makeMenuHTMLDoc()   - 
Method in class util.apps. MakeBioTools 
 Generates the cmdLnMenus.html doc.
   makeMiRNAs(HashMap)   - 
Static method in class expr. ParseMiRNATable 
  
   makeMockIntervals(double)   - 
Method in class trans.main. SetNumberIntervalMaker 
 Returns the number of intervals that can be made at a given cutoff
   makeMockJars()   - 
Method in class edu.utah.seq.misc. MakeUSeq 
 Makes mock Jar files.
   makeMockJars()   - 
Method in class util.apps. MakeBioTools 
 Makes mock Jar files.
   makeMultiFASTA(String, DBUtil)   - 
Static method in class bioroot. Search 
  
   makeNameFileHash(File[])   - 
Static method in class util.gen. IO 
 Returns a HashMap of the file name sans extension and the file.
   makeOligoBeans(DBUtil, UserBean)   - 
Static method in class bioroot. Archive 
 Makes an array of all visibile OligoBeans
   makeOligoConcat(BlastMatcher[])   - 
Static method in class bioroot.plasmid. PlasmidBase 
 Converts a BlastMatcher[] to a concat of oligo sequences and NNNNNNNNNN spacers.
   makePanel(Interval, int, TextFrame, boolean)   - 
Method in class trans.graphics. IntervalDrawFrame 
  
   makePanel(float[][], int, float)   - 
Method in class trans.graphics. VirtualCelFrame 
  
   makePlasmidBeans(DBUtil, UserBean)   - 
Static method in class bioroot. Archive 
 Makes an array of all visibile PlasmidBeans
   makePNG(File, double)   - 
Method in class gata.menu. SaveImagePanel 
  
   makePNGPanel(float[][], int, File)   - 
Method in class trans.graphics. VirtualCelFrame 
  
   makePointData()   - 
Method in class edu.utah.seq.parsers. ElandParser 
 Makes the PointData hash after sorting, writes to disk.
   makePoints(int[], float[])   - 
Static method in class edu.utah.seq.data. Point 
  
   makePositives()   - 
Method in class trans.roc. MethodTester 
  
   makePositives(File)   - 
Method in class trans.roc. MethodTester 
  
   makePositives()   - 
Method in class trans.roc. SgrBacCounter 
  
   makePSPM(String[])   - 
Static method in class meme. MemeMotif 
 This method returns a position specific probability weight matrix given an array
 of Strings, each of the same size, containing GATC, no gaps, no N's.
   makeQPCRWindows(Window[])   - 
Method in class trans.misc. BestWindowScoreExtractor 
  
   makeQuantiles(float[][])   - 
Static method in class trans.cel. QuantileNormalization 
 Makes a Quantile[][] given float[][] where the quantile.position is assigned its
 array index position and the quantile.value is assigned the float value.
   makeRandom(Positive, double)   - 
Method in class trans.roc. ScoreParsedBars 
 Takes a Positive and finds 1000 chrom, length, gc, and number scores matched random regions.
   makeRandomBindingRegions(BindingRegion[], HashMap, int)   - 
Static method in class trans.anno. AnnotateRegions 
 For each binding region this will make another binding region from the same chromosome with the 
 same length, yet at a random location.
   makeRandomBindingRegions(BindingRegion[], HashMap, int)   - 
Static method in class trans.anno. AnnotateRegionsWithGeneList 
 For each binding region this will make another binding region from the same chromosome with the 
 same length, yet at a random location.
   makeRandomRegions(String)   - 
Method in class trans.anno. IntersectRegions 
  
   makeRanked(Coordinate[])   - 
Static method in class util.bio.annotation. ScoredCoordinate 
 Returns an array of ScoredCoordinate where the score is the index position of the Coordinate[].
   makeRankedFloatArrays(ExpressedGene[])   - 
Method in class expr. CorrelationMaps 
 Makes and places RankedFloatArrays in each ExpressedGene,
 also nulls the ExpressedGene.values.
   makeRankedHitsGraph()   - 
Method in class trans.graphics. RankedSetAnalysis 
  
   makeRectangle(int, int, double, double, double, double)   - 
Static method in class gata.main. GATAUtil 
 Returns a rectangle given start stop in nucleotides and y coords in pixels
   makeRectangleAndPoint(boolean, double, double, double, double, Color)   - 
Method in class trans.graphics. GenomicRegionGlyph 
  
   makeRectangles()   - 
Method in class trans.graphics. RankedSetDrawPanel 
  
   makeReference(String[])   - 
Method in class edu.utah.seq.methylation454. ReadAlignParser 
  
   makeRegions(File)   - 
Method in class trans.anno. RandomRegions 
 Parses the regions file but then adds a pointer per base pair length/ divider to
 the Region[] for every region.
   makeSamples()   - 
Method in class edu.utah.seq.methylation454. MethylationParser 
  
   makeSamples(float[], float[])   - 
Method in class trans.main. WilcoxonRankSumTest 
  
   makeSamples(float[][], float[][], int, int)   - 
Method in class trans.main. WilcoxonRankSumTest 
 Builds samples from part of a float[replica][intensities] for treat and control.
   makeScaleBar()   - 
Method in class gata.geneGlyphs. ScaleBar 
 Makes a scale bar between 10 and 100 pixels in length at 1, 10, 100 bp or kb or mb sizes,
        change the scaleBarX and scaleBarY coordinates to relocate the bar
   makeScaleRuler()   - 
Method in class gata.geneGlyphs. ScaleBar 
 Makes a scale ruler with intervals between 10 and 100 pixels in length at 1, 10, 100 bp or kb or mb sizes,
                change the scaleRulerStartStopX and scaleRulerY coordinates to relocate the bar
   makeSegDats()   - 
Method in class trans.misc. CalculateGeneExtensions 
 Segments genes: applies median value across entire exon and intron structure,
 collects intensities surrounding gene,
 runs Hubers segmentation script on parsed gene.
   makeSegments(ArrayList, double, double, double)   - 
Static method in class gata.main. GATAUtil 
 Converts an ArrayList of int[2]'s (startStop) in nucleotides to an ArrayList of Line2D.Double's
   makeSegments()   - 
Method in class util.bio.annotation. Translation 
  
   makeSegmentsInt(int[][], double, double, double)   - 
Static method in class gata.main. GATAUtil 
 Converts an int[x][2]'s (startStop) in nucleotides to an ArrayList of Line2D.Double's
   makeSetNumberEnrichedRegions()   - 
Method in class edu.utah.seq.analysis. EnrichedRegionMaker 
 Will attempt to make the desired number of ERs
   makeSets(File[], File)   - 
Method in class trans.cel. MakeChromosomeSets 
 Merges chromosome info together and saves as whole chromosomes, will also save the oligoPositions if oligoPositionsDirectory isn't null.
   makeSgrFile(Window[], File, int)   - 
Method in class trans.main. ScanChip 
 Makes a xxx.sgr.zip file from a sorted Window[].
   makeShapes(double, double, double, double, double, double, double)   - 
Method in class gata.aligner. Alignment 
  
   makeSlider(JSlider, int, int)   - 
Method in class gata.plotter. Sliders 
  
   makeSource()   - 
Method in class edu.utah.seq.misc. MakeUSeq 
 Makes the code library jar from the directoriesForLibJar array.
   makeSource()   - 
Method in class util.apps. MakeBioTools 
 Makes the code library jar from the directoriesForLibJar array.
   MakeSpliceJunctionFasta   - Class in  util.bio.seq  Generates a fasta file for each.   MakeSpliceJunctionFasta(String[])   - 
Constructor for class util.bio.seq. MakeSpliceJunctionFasta 
  
   makeStrainBeans(DBUtil, UserBean)   - 
Static method in class bioroot. Archive 
 Makes an array of all visibile StrainBeans
   makeSubAligns(LocalAlignment[])   - 
Static method in class gata.main. GATAUtil 
  
   makeSubSeqs()   - 
Method in class selex. SeqRead 
  
   makeSwingComponents()   - 
Method in class util.apps.gump. Gump 
  
   makeSwingComponents()   - 
Method in class util.apps.gump. Option 
  
   MakeTranscriptome   - Class in  util.bio.seq  Generates a fasta file for each gene model containing exon seqs plus splice junctions.   MakeTranscriptome(String[])   - 
Constructor for class util.bio.seq. MakeTranscriptome 
  
   makeTransformer()   - 
Static method in class trans.misc. TransformRatios 
  
   MakeUSeq   - Class in  edu.utah.seq.misc  Utility app to pull classes into folders and extract menus to html.   MakeUSeq()   - 
Constructor for class edu.utah.seq.misc. MakeUSeq 
  
   makeVirtualCel(int[][], int)   - 
Static method in class trans.qc. CoordinateExtractor1lq 
 Make a virtual cel file loading xyCoors with an intensity of 100.
   makeVirtualCel(int[][], int)   - 
Static method in class trans.qc. Parser1lq 
 Make a virtual cel file loading xyCoors with an intensity of 100.
   makeVisableAlignments()   - 
Method in class gata.plotter. AlignPanel 
 Resets an array of visable alignments
   makeWindows()   - 
Method in class edu.utah.seq.analysis. ScanSeqs 
 Makes a common set of windows using merged positions.
   makeWindows(File)   - 
Static method in class trans.misc. BestWindowScoreExtractor 
 Converts a tab delimited txt file of chr, start, stop, scores (multiple permitted or none)
 into an array of Windows.
   makeWindows(int[])   - 
Method in class trans.tpmap. WindowMaker 
 Takes an int[] of sorted bp start positions and finds unique windows of 
 a maximized width with a minimal # of features.
   makeWindowsFromAlign()   - 
Method in class gata.aligner. LocalAlignment 
  
   makeWindowsFromFiles(String[])   - 
Method in class trans.tpmap. WindowMaker 
 Takes int[] files containing bp start positions for each feature, ordered.
   makeXMLReader()   - 
Static method in class util.gen. XML 
 create a new XML reader, returns null if a problem is encountered.
   mapCelFiles(File[])   - 
Method in class trans.cel. CelMapper 
  
   mapCelFiles(File[], File[])   - 
Method in class trans.cel. CelMapper 
 Maps float[][] to MapFeature[][] saving under the index matched File in matchedSaveAsFiles as a MultiSetQuantile[].
   MapControlIntensities   - Class in  trans.bkgrnd  Loads a bpmap file with oligo intensity measurements from multiple '.cel' files after median scaling to 50.   MapControlIntensities()   - 
Constructor for class trans.bkgrnd. MapControlIntensities 
  
   MapFeature   - Class in  trans.tpmap  Tpmap line broken into some components, chromosome, start, pmX, pmY, and optionally, mmX, mmY.   MapFeature(String)   - 
Constructor for class trans.tpmap. MapFeature 
 Constructor - bpmapLine - seq ori chrom start pmX pmY mmX mmY, (mm coords are optional).
   mapIt(Matcher)   - 
Static method in class bioroot. PatternMatch 
 Returns an array of hits (PatternMatch[]) given a Matcher.
   mapItArrayList(Matcher)   - 
Static method in class bioroot. PatternMatch 
 Returns an ArrayList of PatternMatch given a Matcher.
   MapSplitter   - Class in  trans.tpmap  Splits a '.bpmap' text file into different chromosomes, helper script for   TPMapProcessor  .   MapSplitter(MapFeature[], File)   - 
Constructor for class trans.tpmap. MapSplitter 
 For running in combination with BPMapProcessor
   Marker   - Class in  bioroot      Marker()   - 
Constructor for class bioroot. Marker 
  
   MarkerBean   - Class in  bioroot      MarkerBean()   - 
Constructor for class bioroot. MarkerBean 
  
   MarkerBean(int, DBUtil)   - 
Constructor for class bioroot. MarkerBean 
  
   mask(Coordinate[], File)   - 
Method in class trans.anno. SubtractRegions 
  
   mask(int, int)   - 
Method in class trans.main. PeakPicker 
  
   maskBoolean(Gff3Feature[])   - 
Method in class util.bio.annotation. ExportIntergenicRegions 
 Uses a boolean[] to score whether a base is part of an annotation (true) or not (false).
   MaskExonsInFastaFiles   - Class in  util.bio.seq  Takes a UCSC gene table and a directory of xxx.fasta files and replaces the exonic sequence with Xs.   MaskExonsInFastaFiles()   - 
Constructor for class util.bio.seq. MaskExonsInFastaFiles 
  
   maskOutliers(float[][], float)   - 
Static method in class util.gen. Num 
 Runs through float[][] setting any values that exceed the outlierThreshold
 to zero.
   matches(BL2SeqHit[])   - 
Method in class bioroot. BlastMatcher 
  
   matches(Sgr)   - 
Method in class trans.roc. Positive 
  
   matches   - 
Variable in class trans.tpmap. MapFeature 
  
   matches(TPMapFeature, TPMapFeature)   - 
Static method in class trans.tpmap. TPMapDuplicateFilter 
 Only checks coordinates!
   matchIt(boolean)   - 
Method in class bioroot. SearchHit 
  
   matchIt(Pattern)   - 
Method in class util.apps. HouseBean 
  
   matchPicks()   - 
Method in class util.bio.parsers. SynonymMatching 
  
   matScore(float[], float[])   - 
Static method in class util.gen. Num 
 Takes unlogged MAT normalized t-values, sorts, logs and takes a variant of a number stabilized trimmed mean ratio.
   maxFileSize   - 
Static variable in class bioroot. Util 
 Maximum allowable file size for upload
   maxLength(Positive[])   - 
Static method in class trans.graphics. AggregatePlots 
 Finds max length of the Region[], stop-start+1
   maxMedianAbsDiffSubWinTreatment(Interval)   - 
Static method in class trans.main. IntervalReportPrinter 
 Calculate maximum median absolute diff of SubWindow treatment values
   maxPosition(String)   - 
Method in class edu.utah.seq.analysis. QCSeqs 
  
   MD5   - Class in  util.gen  This class implements the MD5 algorithm as described in RFC1321.   MD5()   - 
Constructor for class util.gen. MD5 
  
   MD5Crypt   - Class in  util.gen  This class defines a method,
   crypt()  , which
 takes a password and a salt string and generates an OpenBSD/FreeBSD/Linux-compatible
 md5-encoded password entry.   MD5Crypt()   - 
Constructor for class util.gen. MD5Crypt 
  
   mean(float[])   - 
Static method in class util.gen. Num 
 Averages a float array.
   mean(float[], float[])   - 
Static method in class util.gen. Num 
 Averages two float[]s .
   mean(float[][], float[][])   - 
Static method in class util.gen. Num 
 Averages two float[][]s, assumes a square.
   mean(int[])   - 
Static method in class util.gen. Num 
 Averages a int array.
   mean(double[])   - 
Static method in class util.gen. Num 
 Averages a double array.
   meanDoubles(ArrayList)   - 
Static method in class util.gen. Num 
 Averages an ArrayList of Double objects.
   meanIgnoreNaNInfinity(float[])   - 
Static method in class util.gen. Num 
 Calculates a mean on the non NaN and Infinity floats, return zero if no non zero values were found.
   meanIntegers(ArrayList)   - 
Static method in class util.gen. Num 
 Averages an ArrayList of Integer objects.
   median(double[])   - 
Static method in class util.gen. Num 
 Calculates Median of a sorted double[].
   median(int[])   - 
Static method in class util.gen. Num 
 Calculates Median of a sorted int[].
   median(short[])   - 
Static method in class util.gen. Num 
 Calculates Median of a sorted short[].
   median(float[])   - 
Static method in class util.gen. Num 
 Calculates Median of a sorted float[].
   medianAbsoluteDifference(float[], float[])   - 
Static method in class util.gen. Num 
 Calculates the median absolute difference.
   medianFloatArray(float[][])   - 
Static method in class util.gen. Num 
  
   medianIgnoreZeros(float[])   - 
Static method in class util.gen. Num 
 Calculates the median value of a sorted float[] ignoring leading zero values!
   medianNormalize(float[], float)   - 
Static method in class util.gen. Num 
 Median normalize unsorted array to a given number.
   medianNormalize(double[], double)   - 
Static method in class util.gen. Num 
 Median normalize unsorted array to a given number.
   medianNormalize(float[][], double)   - 
Static method in class util.gen. Num 
 Median normalizes an unsorted float[][] array to a given target.
   medianRatioSubWindow(Interval)   - 
Static method in class trans.main. IntervalReportPrinter 
 Calculate Median ratio of SubWindow where the treatments are averaged, and the controls are averaged
   medianScale(LoadedOligo[])   - 
Static method in class trans.bkgrnd. CalcBkgrndRatios 
  
   medianScaleIntensities()   - 
Method in class trans.cel. QuantileNormalization 
 Median scale current intensity values.
   meme   - package meme Wrapper for meme tweaked for processing multiple rounds of SELEX oligos.   MemeMotif   - Class in  meme  This contains info generated from parsing a meme report, each found motif is represented by this class.   MemeMotif()   - 
Constructor for class meme. MemeMotif 
  
   MemeParser   - Class in  meme  Parses meme output into MemeMotif objects and stores some generalize info re the meme run.   MemeParser(String)   - 
Constructor for class meme. MemeParser 
 For single processor meme
   MemeParser(String, String, String)   - 
Constructor for class meme. MemeParser 
 For parallel processor meme on Sapo
   MemeR   - Class in  meme  Main, wRapper for meme geared toward processing multiple rounds of selex data.   MemeR()   - 
Constructor for class meme. MemeR 
  
   MemeResults   - Class in  meme  Holds the results from various parts of MemeR.   MemeResults()   - 
Constructor for class meme. MemeResults 
  
   menuHeader   - 
Static variable in class edu.utah.seq.misc. MakeUSeq 
  
   menuHeader   - 
Static variable in class util.apps. MakeBioTools 
  
   mergeAndSavePointData()   - 
Method in class edu.utah.seq.analysis. QCSeqs 
  
   mergeClusters(double[])   - 
Method in class util.bio.cluster. HierarchicalClustering 
 Merges Clusters from best pair notes whether they have a bad cc.
   MergeColumns   - Class in  util.apps      MergeColumns()   - 
Constructor for class util.apps. MergeColumns 
  
   MergeFastas   - Class in  util.bio.seq  Merges many fasta's into one fasta with a defined number of X spacers.   MergeFastas(String[])   - 
Constructor for class util.bio.seq. MergeFastas 
  
   mergeFeatures(Gff3Feature[])   - 
Static method in class util.bio.parsers.gff. ParseWormBaseGffToUCSC 
 Uses a boolean[] to score whether a base is part of an annotation (true) or not (false).
   mergeLAArrays(LocalAlignment[], LocalAlignment[])   - 
Static method in class gata.main. GATAUtil 
  
   mergePointData(HashMap<String, PointData>[], boolean, boolean)   - 
Static method in class edu.utah.seq.data. PointData 
 Merges a HashMap  by either summing same position scores or simply concatinating then sorting the arrays.
   mergePointData(ArrayList<PointData>, boolean)   - 
Static method in class edu.utah.seq.data. PointData 
 Merges PointData, nulls originals positions and scores, sets strand to '.' if mixed strand.
   MergeRegions   - Class in  trans.anno  Takes several tab delimited region files (chrom, start, stop, ...), creates the union and writes to disk.   MergeRegions()   - 
Constructor for class trans.anno. MergeRegions 
  
   mergeSubAligns(ArrayList)   - 
Method in class gata.aligner. LocalAlignment 
  
   MergeWindowArrays   - Class in  trans.main      MergeWindowArrays(String[])   - 
Constructor for class trans.main. MergeWindowArrays 
  
   mergeWindows()   - 
Method in class trans.main. T2 
 Creats one or two window merged window arrays containing all of the chromosomes.
   MethodTester   - Class in  trans.roc  Application for scoring '.sgr' files for positives and negatives in relation to known (spike in) data.   MethodTester(String[])   - 
Constructor for class trans.roc. MethodTester 
  
   MethylationParser   - Class in  edu.utah.seq.methylation454      MethylationParser(File)   - 
Constructor for class edu.utah.seq.methylation454. MethylationParser 
  
   millisecToDays(long)   - 
Static method in class util.gen. Num 
 Converts milliseconds to days.
   minMax(SmoothingWindowInfo[], int)   - 
Static method in class edu.utah.seq.data. SmoothingWindowInfo 
 Returns min and max of the scores for a given scoreIndex.
   minMax()   - 
Method in class expr. CorrelationMaps 
  
   minMaxWindowArray(Window[], int)   - 
Static method in class trans.misc. Util 
 Returns the min and max values of a given score index in a Window[].
   minMaxWindowScores()   - 
Method in class trans.main. SetNumberIntervalMaker 
 Finds min max
   minMaxWindowScores(Window[], int)   - 
Static method in class trans.misc. Util 
 Finds min max score
   minus10log10(double)   - 
Static method in class util.gen. Num 
 Returns the -10 * LOG base 10 of the number.
   MiRNA   - Class in  expr      MiRNA(String, MiRNATarget[])   - 
Constructor for class expr. MiRNA 
  
   MiRNATarget   - Class in  expr      MiRNATarget(String, String, String, String)   - 
Constructor for class expr. MiRNATarget 
  
   Misc   - Class in  util.gen  A variety of static methods.   Misc()   - 
Constructor for class util.gen. Misc 
  
   mmX   - 
Variable in class trans.tpmap. MapFeature 
  
   mmY   - 
Variable in class trans.tpmap. MapFeature 
  
   modeOfHistogram(int[])   - 
Static method in class util.gen. Num 
 Finds mode of a int[] histogram array, assumes one peak, returns the index position, value/frequency.
   modifyEnds()   - 
Method in class selex. SelexParams 
  
   modifySequenceEnds(String[], String[], String, String, String, String)   - 
Static method in class selex. SeqFiles 
 Modifies the left side and right side of Sequences also adds (100's) or subtracts quanilty scores to match.
   MotifHit   - Class in  meme  Holds data related to a particular match of a matrix to a sequence.   MotifHit(int, int, double, String, int)   - 
Constructor for class meme. MotifHit 
  
   MotifScanner   - Class in  meme  MotifScanner objects are used to scan sequences with a particular weight matrix and generate 
 an array of MotifHit objects.   MotifScanner(double[][], MemeResults)   - 
Constructor for class meme. MotifScanner 
  
   MotifScanner(double[][])   - 
Constructor for class meme. MotifScanner 
  
   MotifSearchResult   - Class in  meme  Holds the results of searching a multi-fasta seq file with a particular motif.   MotifSearchResult(MemeMotif, String, int)   - 
Constructor for class meme. MotifSearchResult 
  
   MultiAlignPanel   - Class in  gata.plotter      MultiAlignPanel()   - 
Constructor for class gata.plotter. MultiAlignPanel 
  
   MultiFastaParser   - Class in  util.bio.parsers  Extracts sequences and headers out of multi fasta files.   MultiFastaParser(File)   - 
Constructor for class util.bio.parsers. MultiFastaParser 
  
   MultiFastaParser(File, int, boolean)   - 
Constructor for class util.bio.parsers. MultiFastaParser 
  
   MultiFastaParser(String)   - 
Constructor for class util.bio.parsers. MultiFastaParser 
  
   MultiFastaParser()   - 
Constructor for class util.bio.parsers. MultiFastaParser 
  
   MultiFastaParser(File[])   - 
Constructor for class util.bio.parsers. MultiFastaParser 
  
   MultiSetQuantile   - Class in  trans.cel  Class holding info relating to one quantile value.   MultiSetQuantile(byte, int, float)   - 
Constructor for class trans.cel. MultiSetQuantile 
  
   MultiSGRPrinter   - Class in  trans.main  Prints a multi sgr file (chr position val1, val2, val3, ....)   MultiSGRPrinter(String[])   - 
Constructor for class trans.main. MultiSGRPrinter 
  
   MultiWindowIntervalMaker   - Class in  trans.main  Collapses arrays of Windows into an array of Interval based on scores and minimal number of windows that must pass.   MultiWindowIntervalMaker(String[])   - 
Constructor for class trans.main. MultiWindowIntervalMaker 
  
   MummerMapper   - Class in  trans.tpmap  Application to map 1lq files to fasta files.   MummerMapper(String[])   - 
Constructor for class trans.tpmap. MummerMapper 
  
 
 


 
   
  
 
 
 
   
 
   
          Overview     
        Package   
        Class   
        Use   
          Tree     
          Deprecated     
       Index    
          Help     
   
 
 
  
 
 
 

 
  
   PREV LETTER   
   NEXT LETTER    
  
    FRAMES     
   NO FRAMES     
  
    All Classes  ');
  }
  //-->
 
 
    All Classes  
 


  
 
 
  
 

 A   B   C   D   E   F   G   H   I   J   L   M   N   O   P   Q   R   S   T   U   V   W   X   Z
